# Economical Speed and Energetically Optimal Transition Speed Evaluated by Gross and Net Oxygen Cost of Transport at Different Gradients

**DOI:** 10.1371/journal.pone.0138154

**Published:** 2015-09-18

**Authors:** Daijiro Abe, Yoshiyuki Fukuoka, Masahiro Horiuchi

**Affiliations:** 1 Center for Health and Sports Science, Kyushu Sangyo University, Fukuoka, Japan; 2 Faculty of Health and Sports Science, Doshisha University, Kyotanabe, Japan; 3 Division of Human Environmental Science, Mt. Fuji Research Institute, Fujiyoshida, Japan; VIT University, INDIA

## Abstract

The oxygen cost of transport per unit distance (CoT; mL·kg^-1^·km^-1^) shows a U-shaped curve as a function of walking speed (*v*), which includes a particular walking speed minimizing the CoT, so called economical speed (ES). The CoT-*v* relationship in running is approximately linear. These distinctive walking and running CoT-*v* relationships give an intersection between U-shaped and linear CoT relationships, termed the energetically optimal transition speed (EOTS). This study investigated the effects of subtracting the standing oxygen cost for calculating the CoT and its relevant effects on the ES and EOTS at the level and gradient slopes (±5%) in eleven male trained athletes. The percent effects of subtracting the standing oxygen cost (4.8 ± 0.4 mL·kg^-1^·min^-1^) on the CoT were significantly greater as the walking speed was slower, but it was not significant at faster running speeds over 9.4 km·h^-1^. The percent effect was significantly dependent on the gradient (downhill > level > uphill, *P* < 0.001). The net ES (level 4.09 ± 0.31, uphill 4.22 ± 0.37, and downhill 4.16 ± 0.44 km·h^-1^) was approximately 20% slower than the gross ES (level 5.15 ± 0.18, uphill 5.27 ± 0.20, and downhill 5.37 ± 0.22 km·h^-1^, *P* < 0.001). Both net and gross ES were not significantly dependent on the gradient. In contrast, the gross EOTS was slower than the net EOTS at the level (7.49 ± 0.32 vs. 7.63 ± 0.36 km·h^-1^, *P* = 0.003) and downhill gradients (7.78 ± 0.33 vs. 8.01 ± 0.41 km·h^-1^, *P* < 0.001), but not at the uphill gradient (7.55 ± 0.37 vs. 7.63 ± 0.51 km·h^-1^, *P* = 0.080). Note that those percent differences were less than 2.9%. Given these results, a subtraction of the standing oxygen cost should be carefully considered depending on the purpose of each study.

## Introduction

It has been well acknowledged that walking and/or running economy can be described as the oxygen cost of transport per unit distance (CoT; mL·kg^-1^·km^-1^) [1–2]. There is a U-shaped relationship between CoT and gait speeds during walking [1–7] and a linear relationship during running [1]. This indicates that there is a particular gait speed minimizing the CoT in each individual during walking, and this gait speed is known as the economical speed (ES) [4–7] or optimal speed [8–12]. In calculations of the CoT, the role of the standing oxygen cost (standing *V*O_2_; mL·kg^-1^·min^-1^) has been argued [3,4,6,7,13]. Many previous studies have found that people preferred walking speeds at or near the gait speed associated with their ES calculated by the total (gross) *V*O_2_ [2,3]. The standing *V*O_2_ accounted for 60% of the total *V*O_2_ at slower gait speeds [13].

Several studies have employed gross *V*O_2_ [2–7,11,12,14,15] and others used the net *V*O_2_ (gross *V*O_2_—standing *V*O_2_) to calculate the net CoT values during walking [8–10,16–24] and running [25–27]. It is reasonable that the net *V*O_2_ has been used when calculating the mechanical efficiency of walking or running at each particular gait speed, because the metabolic rate derived from the body movement itself can be evaluated. The individual ES or preferred walking speed could be related to the maximal recovery of the mechanical energy [16,17,19,28,29], but it is interesting to note that the net ES was always slower than the gross ES [10,11,17–19,28,30].

The CoT-*v* relationship has been known to be approximately constant during running [26,27], meaning that there is an intersection at which the U-shaped CoT-*v* relationship (for walking) and linear CoT-*v* relationship (for running) in each individual, so called *energetically optimal transition speed* (EOTS) [31–33]. An individual gait transition from walking to running is not necessarily determined by the metabolic demands [32–35]. Biomechanical and/or anthropometric mechanisms explaining the gait transition have been argued [36–42], but this matter is still controversial. The standing *V*O_2_ in non-obese young male adults has been reported to be approximately 4–5 mL·kg^-1^·min^-1^ [21,26,27], meaning that the effect of subtracting the standing *V*O_2_ on the CoT-*v* relationship must be relatively greater at slower gait speeds. This is because the oxygen cost is lesser at slower gait speeds. To the best of our knowledge, there are few reports regarding the effects of subtracting the standing *V*O_2_ on both CoT-*v* relationships and the relevant effect on the ES or EOTS at different gradients. It was hypothesized that the CoT-*v* relationship after subtracting the standing *V*O_2_ could present a possible combination of the leftward (slower) shift for walking and a downward shift for running. Such possible alterations in both CoT-*v* relationships would result in the slower net ES and EOTS. The purpose of this study was to investigate the effects of subtracting the standing *V*O_2_ on the calculation of the CoT and the relevant effects of this subtraction on the ES or EOTS on a level surface and on gradient slopes.

## Methods

### Participants

Eleven male well-trained runners (7 distance runners = 5 km best record within 16’51” and 4 sprinters = 100 m best record within 12”0) participated in this study. The mean age, body height, and body mass were 19.8 ± 1.0 years old, 1.704 ± 0.058 m, 58.9 ± 6.2 kg. A written informed consent was obtained from all participants after detailed explanations of all procedures, purpose of this study, possible risks, and benefits of the participation. This study conformed to the Declaration of Helsinki, and an ethical committee established in Kyushu Sangyo University approved the purpose and all procedures of this study (H240324 and H27-0002 as an updated approval).

### Exercise protocols and measurements

All studies were carried out on a motor-driven treadmill (LABORDO LXE1200, Senoh, Japan). The participants wore underwear, shirts, socks, shorts and lightweight training shoes [4,6,7]. The treadmill gradient was set at 0% (level), -5% (downhill), and +5% (uphill) under consideration of daily activities [4,6,7]. The standing oxygen consumption (*V*O_2_; mL·kg^-1^·min^-1^) was preliminarily measured in eight different participants at each of the gradient with a randomized order, and no significant differences were found among the standing *V*O_2_ at those different gradients (4.81 ± 0.50, 4.75 ± 0.50, and 4.82 ± 0.36 mL·kg^-1^·min^-1^ for the level, uphill, and downhill gradients, respectively). Thus, the standing *V*O_2_ was measured on the first day of a series of measurements. The subjects sat on a chair for 5 minutes with a gas collection mask. The participants stood up on a flat terrain for 5–6 minutes. The average oxygen consumption for the last 2-min was regarded as the standing *V*O_2_.

To be accustomed to the treadmill walking and running, each participant walked and run at least three preliminary practices on the treadmill with a freely chosen step frequency at several gait speeds and gradients. Eight gait speeds were incrementally set at 2.4, 3.1, 3.8, 4.5, 5.2, 5.9, 6.6, and 7.3 km·h^-1^ for walking and four gait speeds at 8.7, 9.4, 10.1, and 10.8 km·h^-1^ for running. Each gait speed lasted for 4 minutes. Between walking and running tests, the participants sat on a chair for 6–7 min. The *V*O_2_ was measured with a computerized breath-by-breath system (AE-310S, Minato Ltd., Osaka, Japan). The standard known gases (O_2_ 15.22%, CO_2_ 5.17%, and N_2_ 79.61%) and room air were used for the calibration of gas analyzer. A single sample of the final 2-min *V*O_2_ value at each gait speed was calculated to obtain the CoT. The order of gradient was randomized, and the participants completed one of these gradient trials in a day. Each particular gross CoT was determined by the ratio of the *V*O_2_ to the gait speed (*v*; m·min^-1^):

$$gross\;CoT\;\left(mL\cdot kg^{-1}\cdot km^{-1}\right)\;=\;\frac{\text{gross }V{\text{O}}_{2}}{v}\;\times \;1000\;\;.$$ (1)

The net CoT was determined as follows:

$$net\;CoT\;\left(mL\cdot kg^{-1}\cdot km^{-1}\right)\;=\;\frac{\text{gross }V{\text{O}}_{2}\;-\text{ standing }V{\text{O}}_{2}}{v}\;\times \;1000.$$ (2)

In human walking, a relationship between gross or net CoT and gait speeds can be approximated with a quadratic equation [1,3–7,9–12,14–20], and it is described as follows:

$$CoT\;\left(v\right)\;=av^{2}+bv+c\;.$$ (3)

Where the coefficients *a*, *b*, and *c* are determined by the least squares regression with data obtained from eight walking speeds. A differential function of Eq 3 can be described as follows:

CoT′ (v) = 2av+b . (4)

Then, the individual ES was determined at the gait speed when the CoT′ (*v*) equals zero, indicating that it can be observed using a following equation:

$$ES\;\left(km\cdot h^{-1}\right)\;=\;\frac{\left|-b\right|}{2a}\;\times \;60\;\times \;\frac{1}{1000}.$$ (5)

In Eq 5, the coefficient *b* is always a negative value, so that the absolute value (|-*b*|) is regarded as the coefficient *b*. Eq 5 indicates that the ES is independent of the coefficient *c*. However, the coefficient *c* would relate to the standing *V*O_2_, because it reflects the *y*-intercept of the CoT-*v* relationship. A standard form of a quadratic equation used in previous studies [43,44] were modified with adopting the first component, and it was applied for a relationship between gross *V*O_2_ per body mass and square speed (m^2^·sec^-2^). Its relationship was approximated as follows:

$$VO_{2}=Ax^{2}+Bx+C\;.$$ (6)

Our present study considered the second component (*Bx*) to reduce residual error, and this is a modified point. Where the coefficients *A*, *B*, and *C* are the coefficients of each component and *x* is the square speeds. A finite difference between coefficient *C* and standing *V*O_2_ was regarded as the second compartment [43,44].

In human running, a relationship between gross or net CoT and gait speeds was approximated using a linear regression analysis, then the CoT during running can be described as follows:

CoT (v) =pv+q . (7)

Where the coefficients *p* and *q* are determined by the least squares regression with data obtained from four running speeds. An intersection (EOTS) between U-shaped quadratic equation and linear regression line is obtained when Eqs 3 and 7 are equal. Rearranging Eqs 3 and 7,

$$av^{2}+\;\left(b-p\right)v+\;\left(c-q\right)\;=\;0.$$ (8)

Previous studies have used preferred transition speed (PTS) instead of EOTS, however, some of them argued that the PTS might be influenced by the protocol [32,36–40]. There is any justification whether the recently proposed criteria [36] are available at different gradients. Thus, the EOTS at each gradient was only used in this study. A following quadratic formula (Eq 9) gives two solutions, and then only a faster one is regarded as the EOTS.

$$EOTS\;\left(km\cdot h^{-1}\right)\;=\;\frac{-(b-p)\pm \;\sqrt{{\left(b-p\right)}^{2}\;-\;4a(c-q)}}{2a}\;\times \;60\;\times \;\frac{1}{1000}.$$ (9)

For investigating the percent effects of subtracting the standing *V*O_2_, a following equation was used:

$$Percent\;effect\;\left(\%\right)\;=\;[1\;-\frac{\text{gross }V{\text{O}}_{2}\;-\text{ standing }V{\text{O}}_{2}}{\text{gross }V{\text{O}}_{2}}]\;\times \;100\;.$$ (10)

### Statistics

Data were presented as mean ± standard deviation (SD). A regression analysis using a quadratic equation was applied to the CoT-*v* relationship for walking. A linear regression analysis was applied to the CoT-*v* relationship for running. The gross CoT, net CoT, and coefficient values (*a*, *b*, and *b-p*) of the Eqs 5 and 9 obtained from each gradient were compared using a three-way repeated measures analysis of variance (ANOVA) within participants (CoT × gradient × speed) using online software (ANOVA 4). A two-way repeated measures ANOVA was applied for comparisons of those percent differences (ratio of net to gross values) among three gradients (3 ratios × 3 gradients). The net and gross ES or EOTS was also compared using two-way repeated measures ANOVA (net/gross × 3 gradients). If a significant *F* value was obtained on the dependent variables, Ryan’s *post hoc* test was applied to the appropriate data sets to detect significant mean differences. Its statistical power has been reported to be equivalent to Tukey’s *post hoc* test [45], and it can be used regardless of the data distribution [45]. A Pearson’s regression analysis was applied for relationships between coefficient values (*a*, *b*, and *b-p*) and ES or EOTS and between coefficient *c* and standing *V*O_2_. The statistical significance was set at the 0.05 probability level.

## Results

### Cost of transport (CoT)

**Fig 1:**
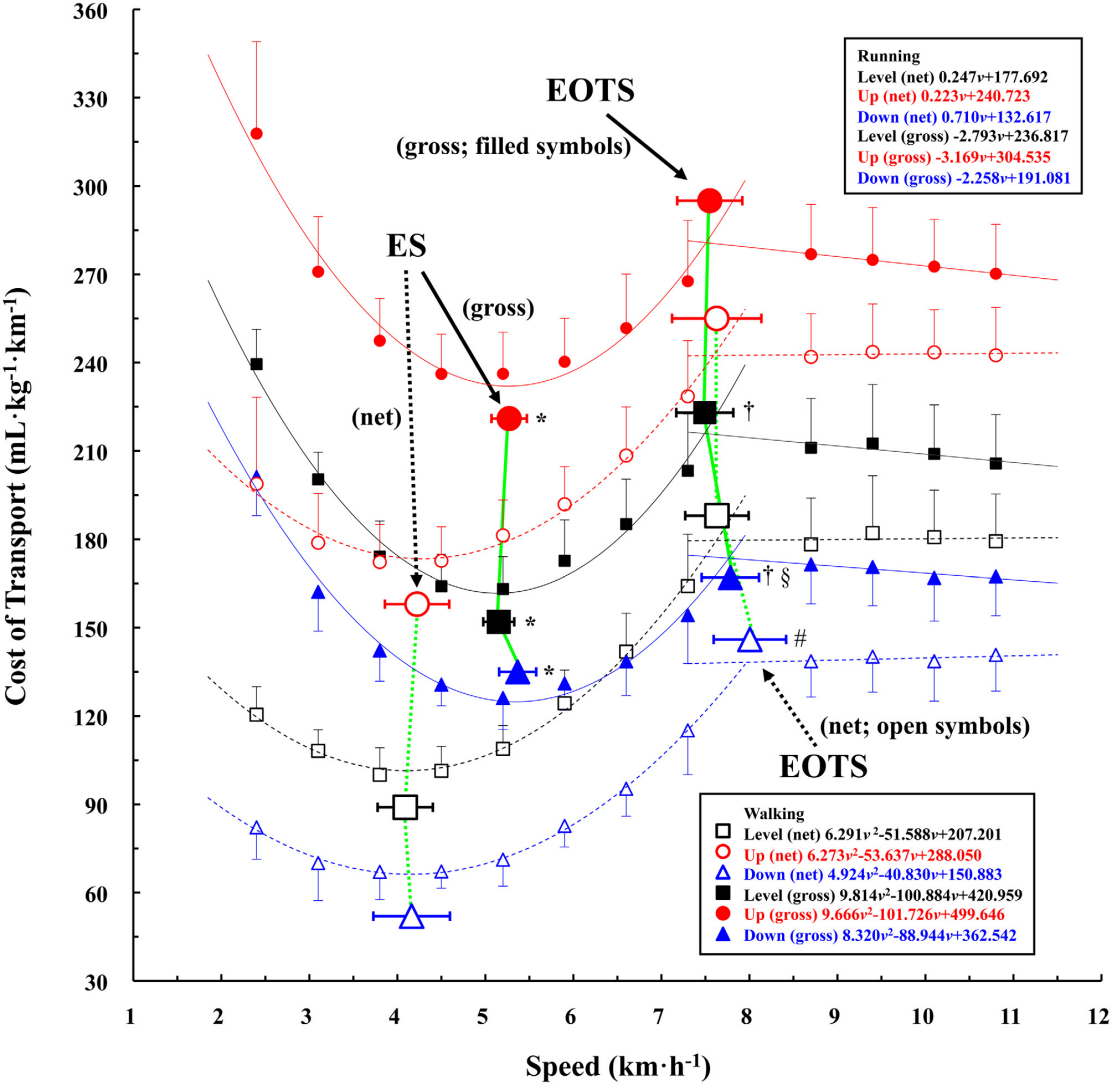
U-shaped and linear cost of transport (CoT) relationships at different gradients. Filled and open symbols show the net and gross CoT, respectively. Circles, squares, and triangles show uphill, level, and downhill gradients, respectively. The mean gross ES, net ES, gross EOTS, and net EOTS are presented. Asterisks (*) indicate ‘gross > net’. Daggers, (†) indicate ‘gross < net’ at the level and downhill gradients. Hash mark (#) indicates ‘faster than the level and uphill gradients’. Section mark (§) indicates ‘faster than level gradient. The ES and EOTS markers were not put based on the correct CoT values, because the markers are likely to overlap with the CoT plots and regression lines. Data are mean ± SD.

The net CoT was significantly lower than the gross CoT at all gait speeds at each gradient (*P* < 0.001, Fig 1). In addition, both gross and net CoT was significantly greater in the order of uphill, level, and downhill gradient at all gait speeds (*P* < 0.001, Fig 1). The mean coefficients (*a*, *b*, and *b-p*) of Eqs 5 and 8, being substantially related to a determination of the ES or EOTS, are summarized in Table 1. The coefficients *a*, *b*, and *b-p* were significantly lesser for the net equations than those of the gross equations (*P* < 0.001, Table 1), while there were no significant gradient differences in those coefficients (*P* = 0.103). The percent ratio of the net to gross equations was significantly lesser in the coefficient *b* and *b-p* than the coefficient *a* (*P* < 0.001, Table 2) without any significant differences among gradients (*P* = 0.066).

**Table 1:** Coefficients *a*, *b*, and *b-p* appeared in Eqs 3–9.

		*a*	*b*	*b-p*
**Level**	**gross**	9.814 ± 1.435*	100.884 ± 13.122*	103.677 ± 12.599*
	**net**	6.291 ± 1.124	51.588 ± 10.875	51.341 ± 10.282
**Uphill**	**gross**	9.666 ± 2.045*	101.726 ± 21.622*	104.895 ± 20.030*
	**net**	6.273 ± 1.976	53.637 ± 20.933	53.286 ± 19.115
**Downhill**	**gross**	8.320 ± 1.519*	88.944 ± 14.601*	90.521 ± 16.229*
	**net**	4.924 ± 1.452	40.830 ± 13.949	40.119 ± 14.730

Coefficients *b* and *b-p* is presented as an absolute value.

* indicate gross > net. Values are mean ± SD.

**Table 2:** Ratio of the net to gross values of each coefficient appeared in Eqs 3–9.

	*a*	*b*	*b-p*
**Level**	63.87 ± 4.05	50.74 ± 5.73*	49.17 ± 5.80*
**Uphill**	63.76 ± 6.48	51.27 ± 8.43*	49.61 ± 8.04*
**Downhill**	58.06 ± 7.32	44.74 ± 8.79*	43.05 ± 9.28*

* indicates *a* < *b* and *b-p*. No gradient difference was found. Values are mean (%) ± SD.

**Fig 2:**
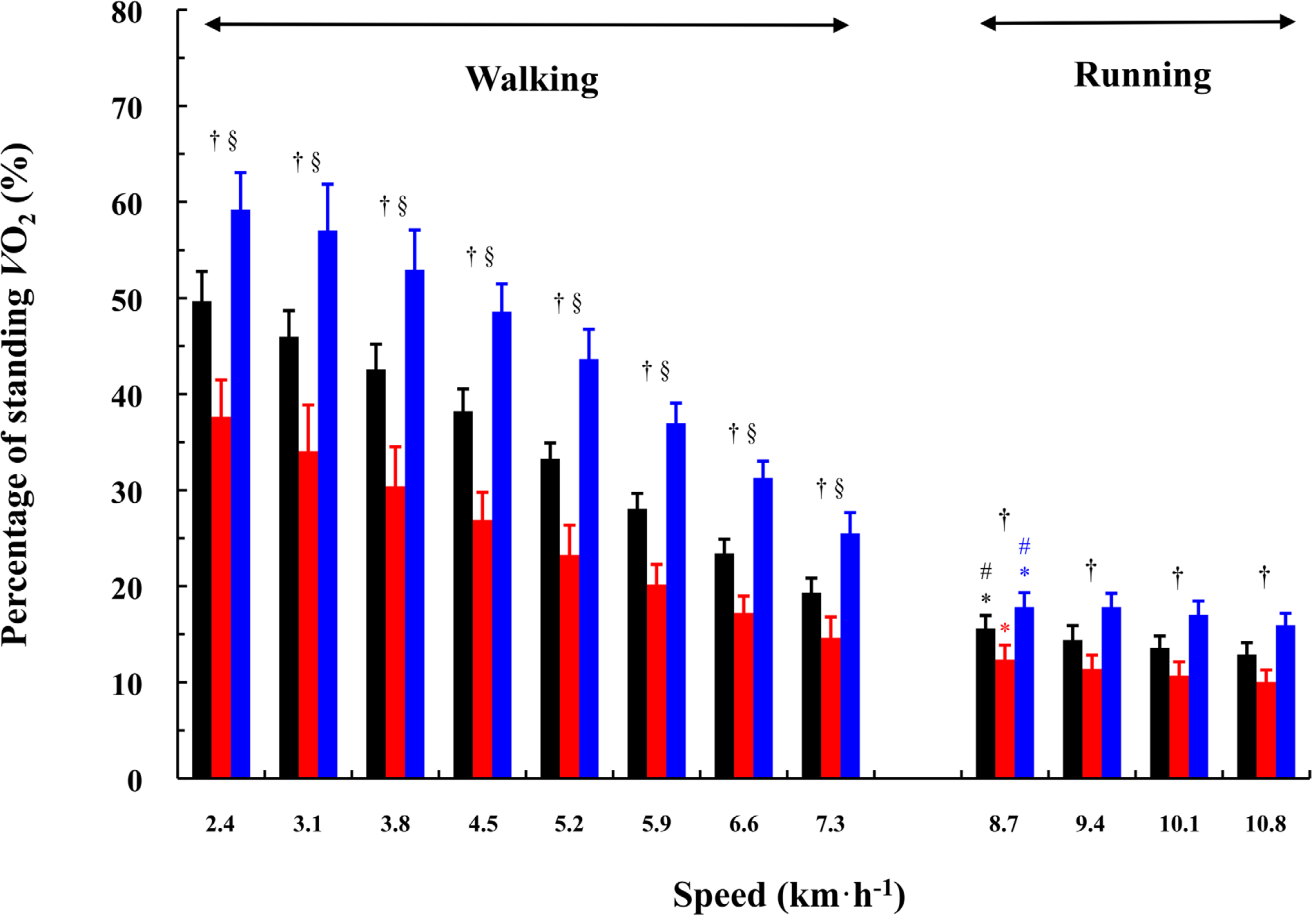
Percentage of the standing *V*O_2_ as a function of gait speed at different gradients. Significant gradient differences were observed at all speeds (†; downhill > level > uphill). There were significant speed-dependent differences in the percent standing *V*O_2_ compared to other speeds (§). There was no significant speed-dependent difference in the percent effects of subtracting the standing *V*O_2_ over 9.4 km·h^-1^. Black, red, and blue bars indicate level, uphill, and downhill gradients, respectively. Values are mean ± SD.

The percent effect of subtracting the standing *V*O_2_ (4.8 ± 0.4 mL·kg^-1^·min^-1^) was significantly greater in the order of downhill, level, and uphill gradient at all gait speeds († marks in Fig 2, downhill > level > uphill, *P* < 0.001). It was also significantly greater as the walking speed was slower (§ marks in Fig 2, *P* < 0.001). Such significant differences were not observed at faster running speeds over 9.4 km·h^-1^ (Fig 2).

**Fig 3:**
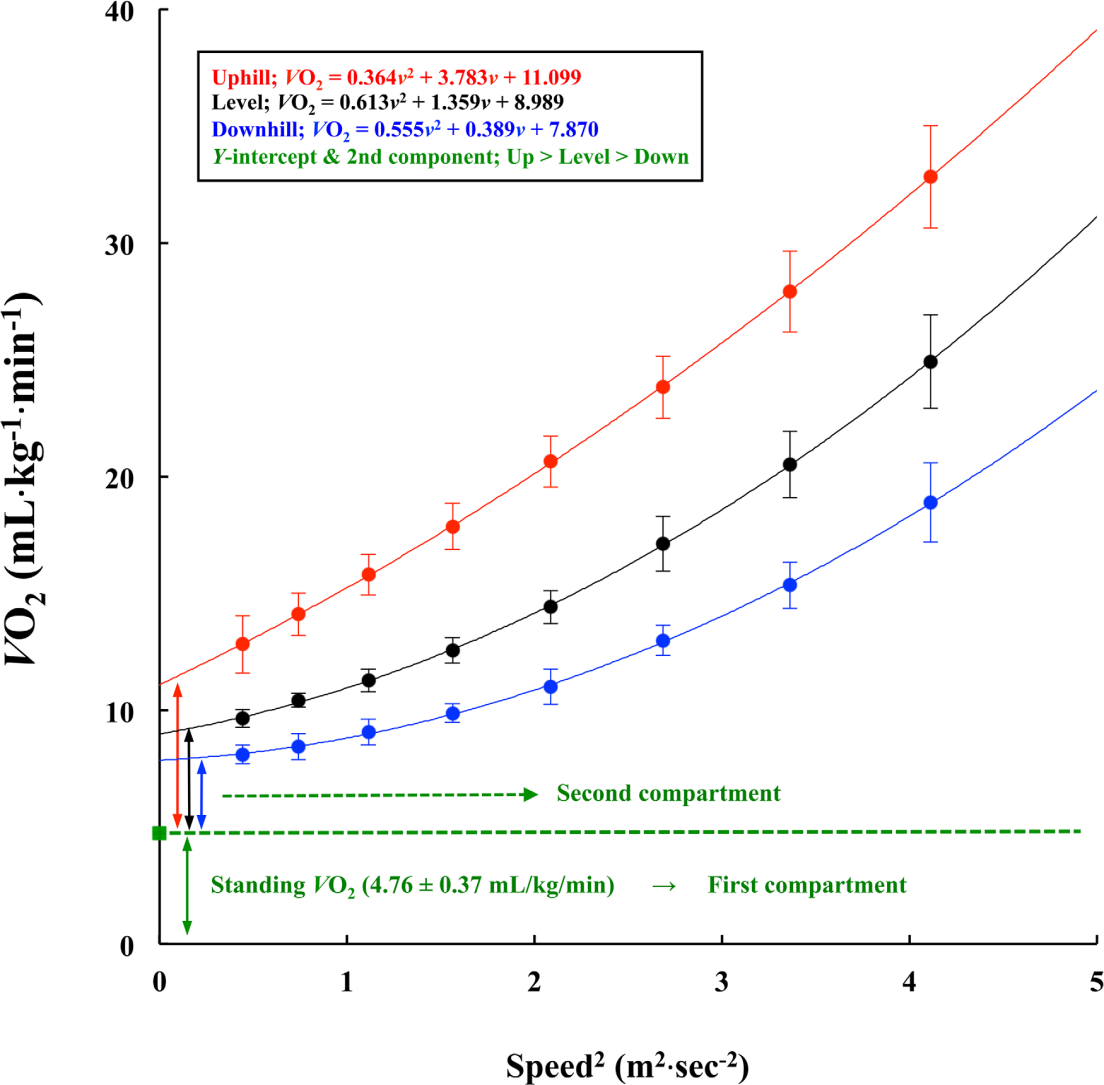
Relationship between *V*O_2_ per body mass and square speed at different gradients. The standing *V*O_2_ is regarded as the first compartment and a finite difference between *y*-intercept (coefficient *C* of Eq 6) and standing *V*O_2_ is regarded as the second compartment. Values are mean ± SD.

The second component, which is a finite difference between standing *V*O_2_ and *y*-intercept, was significantly greater in the order of uphill (6.3 ± 1.1 mL·kg^-1^·min^-1^), level (4.2 ± 0.7 mL·kg^-1^·min^-1^), and downhill (3.1 ± 0.8 mL·kg^-1^·min^-1^) gradient (*P* < 0.001, Fig 3).

### Economical speed (ES)

The CoT-*v* relationships obtained from eight walking speeds were well approximated using Eq 3 (*r* = 0.983–0.999, Fig 1). The mean gross ES values were 5.152 ± 0.176 km·h^-1^ for the level gradient, 5.271 ± 0.201 km·h^-1^ for the uphill gradient, and 5.367 ± 0.221 km·h^-1^ for the downhill gradient (Fig 1). The mean net ES values were 4.089 ± 0.314 km·h^-1^ for the level gradient, 4.224 ± 0.365 km·h^-1^ for the uphill gradient, and 4.162 ± 0.435 km·h^-1^ for the downhill gradient, respectively (Fig 1). These net ES values were significantly slower than the gross ES at all gradients (*P* < 0.001, Fig 1). The percent differences between gross and net ES were 20.2%, 19.9%, and 22.5% at the level, uphill, and downhill gradients, respectively. No significant gradient differences were observed between gross and net ES. Note that the lesser the coefficient *a* or the greater the coefficient *b*, the faster the ES (Eq 5), however, the coefficient *a* alone showed a significant relationship at the downhill gradient for the gross ES only (Table 3, *P* < 0.05). The coefficient *b* alone showed significant relationships at the level and uphill gradients for the net ES (Table 3, *P* < 0.05), but not at the downhill gradient (Table 3).

**Table 3:** Correlation coefficients and *p* values of each relationship.

		gross ES	net ES	gross EOTS	net EOTS
**Level**	*a* (gross)	*r* = -0.508 (0.110)	-	*r* = ***-0*.*677 (0*.*022)***	-
	*b* (gross)	*r* = -0.297 (0.376)	-	-	-
	*a* (net)	-	*r* = +0.526 (0.096)	-	*r* = ***-0*.*674 (0*.*023)***
	*b* (net)	-	*r* = ***+0*.*709 (0*.*015)***	-	-
	*b-p* (gross)	-	-	*r* = -0.583 (0.060)	-
	*b-p* (net)	-	-	-	*r* = -0.467 (0.148)
**Uphill**	*a* (gross)	*r* = -0.221 (0.514)	-	*r* = ***-0*.*681 (0*.*021)***	-
	*b* (gross)	*r* = -0.040 (0.907)	-	-	-
	*a* (net)	-	*r* = +0.540 (0.086)	-	*r* = ***-0*.*625 (0*.*040)***
	*b* (net)	-	*r* = ***+0*.*690 (0*.*019)***	-	-
	*b-p* (gross)	-	-	*r = -0*.*663 (0*.*026)*	-
	*b-p* (net)	-	-	-	*r* = -0.549 (0.080)
**Downhill**	*a* (gross)	*r* = ***-0*.*625 (0*.*040)***	-	*r* = -0.588 (0.057)	-
	*b* (gross)	*r* = -0.493 (0.124)	-	-	-
	*a* (net)	-	*r* = +0.385 (0.242)	-	*r* = ***-0*.*641 (0*.*034)***
	*b* (net)	-	*r* = +0.560 (0.073)	-	-
	*b-p* (gross)	-	-	*r* = -0.502 (0.058)	-
	*b-p* (net)	-	-	-	*r* = -0.537 (0.089)

Bold italic numbers indicate significant correlations (*p* < 0.05). Non-italic numbers indicate *p* > 0.05 (n.s.). Thin italic number indicates *p* < 0.05, but it is an opposite tendency toward faster EOTS, respectively.

### Energetically optimal transition speed (EOTS)

The mean gross EOTS values were 7.493 ± 0.324 km·h^-1^ for the level gradient, 7.550 ± 0.370 km·h^-1^ for the uphill gradient, and 7.783 ± 0.326 km·h^-1^ for the downhill gradient (Fig 1). The mean net EOTS values were 7.631 ± 0.360 km·h^-1^ for the level gradient, 7.628 ± 0.507 km·h^-1^ for the uphill gradient, and 8.005 ± 0.412 km·h^-1^ for the downhill gradient, respectively (Fig 1). The percent differences between gross and net EOTS were 1.8%, 1.0%, and 2.9% at the level, uphill, and downhill gradients, respectively. The gross EOTS was significantly slower than the net EOTS at the level († mark, *P* = 0.003) and downhill gradients († mark, *P* < 0.001), but not at the uphill gradient (*P* = 0.080). The gross EOTS at the downhill gradient was significantly faster than that at the level gradient (§ mark, *P* = 0.001). The net EOTS at the downhill gradient was significantly faster than those at the level and uphill gradients (# mark, *P* < 0.001).

The coefficient *a* was significantly related to the gross and net EOTS (Table 3, *P* < 0.05) except at the downhill gradient for the gross EOTS (Table 3, *P* = 0.057). In contrast, the coefficient *b-p* was not significantly related to the EOTS except at the uphill gradient (Table 3, *P* = 0.026), although it was a negative trend for faster EOTS.

## Discussion

### Cost of Transport (CoT)

Little information has been available with regard to the coefficient behavior of approximating equations listed in the methodological section above. For instance, the coefficient *c* of Eq 3 might be related to the standing *V*O_2_ as explained before. However, there were no significant relationships between the coefficient *c* alone and standing *V*O_2_ (*r* = 0.417 at the level, *r* = 0.304 at the uphill, and *r* = 0.368 at the downhill gradients, respectively). Eq 6 explains the physiological associations of the standing *V*O_2_ and the coefficient *c*. This model was used previously to explain different metabolic rates before and after successive weight loss in obese adolescents [43] or a greater CoT values in elderly people [44]. Peyrot et al. [43] showed that the *y*-intercept of the relationship between the metabolic rate and square speed was significantly decreased after successive weight loss due to a combination of 12-week nutritional guidance and exercise training. Note that a slight downward shift of the CoT-*v* relationship was observed after successive weight loss [43], but the gross ES was not altered due to such interventions in adolescents. These results indicated that the shape of the U-shaped CoT-*v* curve remained unchanged after successive weight loss. This concept was applied for comparing the data at different gradients. The second compartment presented in Fig 3 has been regarded as the oxygen cost in association with maintaining balance and supporting the body mass [43,44]. There was a significant difference in the second compartment (coefficient *C* of Eq 6) among three gradients (uphill > level > downhill, Fig 3), indicating that the oxygen cost for balancing the gait was significantly different among three gradients. Summarizing the above findings, the coefficient *c* of Eq 3 would reflect the sum of the standing *V*O_2_ and oxygen cost for supporting and balancing the body mass.

There are several identical examples to use the net CoT when comparing different gait styles [24,46,47], species [48,49], or body dimensions [10,18,20]. Age comparison might be also included, because elderly people exhibit distinctive gait characteristics [18,21–23,50]. Nevertheless, a practical consideration is necessary. As stated before, the net *V*O_2_ could be better when calculating the mechanical efficiency at each particular gait speed, because the metabolic rate derived from the body movement itself can be evaluated. However, when comparing the oxygen cost obtained from different conditions, the net CoT, rather than the gross CoT, tends to detect statistically significant differences, particularly at slower gait speeds. This is because the percent effects of subtracting the standing *V*O_2_ are greater when the metabolic rate is low (Fig 2). Indeed, some previous studies measured the CoT at one walking speed only [20,23,24]. Peyrot et al. [20] actually measured the CoT values at several walking speeds, but they chose the representative speed, which almost corresponded to the preferred walking speed. Castillo et al. [24] did the similar way. In contrast, Hortobágyi et al. [23] measured the net CoT only at 3.53 km·h^-1^, which was much slower than the preferred walking speed. This must be because the CoT was measured not only at the level gradient but also at the uphill gradient in elderly adults [23]. These previous findings indicated that a practical handling either the gross or net *V*O_2_ for calculating the CoT values should be carefully considered to interpret the phenomenon.

The CoT-*v* relationship for running is substantially related to the running economy, which has been regarded as one of the determinant factors for a success of running events [26,51,52]. As shown in Fig 1, our present study found slightly negative linear trends in the gross CoT-*v* relationships in running at all gradients. A utilization of the stored elastic energy in the muscle-tendon complex has been acknowledged to function as one of the energy-saving mechanisms during running [1,28,29,51]. The strained energy in the human foot arch and its connecting tissues also serve as the energy-saving mechanism [53]. The running speeds tested were set until 10.8 km·h^-1^ in our present study, assuming that a slightly negative linear CoT-*v* relationship in running might be explained by a minimization of the leg muscle activities [54].

### Economical Speed (ES)

It is worth noting that the net ES values were approximately 20% slower than the gross ES values at all gradients (Fig 1), indicating that the CoT-*v* relationship leaned to the leftward (i.e., a slower shift) at all gradients. Such a difference is in line with the result of some previous studies at the level gradient only [11,12]. In this matter of the slower net ES at the level gradient, our hypothesis was supported.

Behaviors of the coefficients *a* and *b* have some physiological implications. First, as explained above, the lesser the *a* or the greater the *b*, the faster the ES. A significantly lesser coefficients *a* and *b* values were found in the net equations than in the gross equations at each gradient (*P* < 0.001, Table 2), indicating that the net ES was expected to be equivalent to the gross ES. However, a greater percent decrease in the coefficient *b* was found than the coefficient *a* (Table 2), resulting that a denominator of Eq 5 became relatively greater when the standing *V*O_2_ was subtracted. Second, the greater the *a* and/or *b*, the wider the shape of the U-shaped CoT-*v* relationship. It allows flexibility for minimizing the CoT at relatively wider range of gait speed around the ES [55]. A non-significant gradient difference was observed in both coefficients (Table 1), suggesting that a gradient difference used in this study (±5%) does not influence the shape of the CoT-*v* relationship.

Another surprising finding of the present study was that a gradient difference was not observed in the net or gross ES (Fig 1), indicating that the gross ES in young trained adults was not influenced by the gradient (±5%). Such a vertical shift without a lateral (slower/faster) shift of the U-shaped CoT-*v* relationship has been reported by Ardigò et al. [56] when walking at more than 10% gradients. Aging [2,15,44,51], but not weight loss by a combination of exercise training and nutritional guidance [43], has been recognized as a potential factor for explaining the upward shift of the U-shaped CoT-*v* relationship. It should be noted that the ES was slowed if a load was carried [3,4,17] or pushed [6]. Different experimental conditions using either gross or net ES might be associated with such a discrepancy.

The standing *V*O_2_ amounted approximately 50% of the absolute *V*O_2_ at the level gradient and 60% at the downhill gradient at 2.4 km·h^-1^ (Fig 2), being supported by a previous study [13]. Such a percent effect of subtracting the standing *V*O_2_ was dependent on the gradient and gait speeds (Fig 2). The preferred walking speed corresponded to the gross ES [2,3,5,10–12,14,43,44]. These findings suggested that the gross CoT should be used with special reference to the calculation of the ES.

### Energetically optimal transition speed (EOTS)

A gait transition necessarily occurs in the bipedal locomotion when the gait speed is increased [31–42,48,49,57]. It is important to note that a smaller percent difference was observed between gross and net EOTS at each of the three gradients tested (< 2.9%, Fig 1). An abrupt increase in electromyography (EMG) activity, particularly in the *tibialis anterior* (TA), has been regarded as a trigger of the gait transition [38–41]. If so, then the uphill gradient is expected to require more TA activity than the level gradient. However, the gross EOTS at the level gradient was not significantly faster than that at the uphill gradient (Fig 1). Indeed, as shown in Fig 1, the U-shaped CoT-*v* relationships leaned to the leftward (slower) when the standing *V*O_2_ was subtracted at all gradients, however, the net EOTS was not significantly slower than the gross EOTS. Conversely, the net EOTS became significantly faster than the gross EOTS at the level and downhill gradients († marks in Fig 1).

The effects of subtracting the standing *V*O_2_ on the EOTS were entirely different from a calculation of the ES, indicating that our hypothesis was entirely rejected in this matter. For determining the EOTS, changes in the coefficients *a* and *b-p* due to the subtraction of the standing *V*O_2_ were almost equivalent to those of the ES (Tables 1 and 2). A discrepancy against the ES was found that the coefficient *a* alone was significantly correlated with the gross and net EOTS at most gradients except for the downhill gradient for the gross EOTS (Table 3).

Many previous studies used PTS, instead of EOTS, with their own protocol for its determination [31–42]. Four objective criteria for determining the PTS were recently proposed [36], and it is impractical to satisfy all criteria for determinations of the individual PTS. It remains unclear whether these criteria are available at different gradients, because gait kinematics differs at different gradients [50]. Note that the PTS was approximately 6% slower than the gross EOTS at the level gradient [31–33]. When running and walking at a 6% slower speed than the EOTS, the *V*O_2_ should be 2.3, 2.1, and 1.5 mL·kg^-1^·min^-1^ greater if running at the level, uphill, and downhill gradients, respectively (see approximations in Fig 1). It is questionable whether humans can perceive such a small metabolic difference when walking or running at the freely chosen step frequency.

A gait speed around the EOTS is less often used in human locomotion [57]. In fact, humans can select either walking or running around the EOTS, although birds feasible for legged bipedal locomotion can neither walk nor run around the gait transition speed [48,49]. In human bipedal locomotion, the PTS does not necessarily match the EOTS [31–34], however, the PTS corresponded to the EOTS in well-trained racewalkers [58]. The EOTS should be regarded as an optimizing phase for faster gait speed (running), which will result in a minimization of the CoT.

## Conclusions

Summarizing these results and those of previous studies, both net and gross *V*O_2_ are available when comparing the CoT values obtained at each gait speed. However, it should be noted that the net CoT at slower gait speeds is likely to detect statistically signify differences. The gross CoT should be used when calculating the ES, resulting that the EOTS had better be calculated using the gross CoT, although the net and gross EOTS did not make much of a difference at each gradient. In contrast, the net CoT should be used for calculating the efficiency of the body movement itself. A subtraction of the standing *V*O_2_ should be carefully handled depending on the purpose of each study.

## Supporting Information

S1 Table Average CoT values at each gradient. (TIF)
